# Human Gaze Following Response Is Affected by Visual Acuity

**DOI:** 10.1155/2014/543478

**Published:** 2014-04-06

**Authors:** Marcella Spoor, Behdokht Hosseini, Bart van Alphen, Maarten A. Frens, Jos N. van der Geest

**Affiliations:** ^1^Department of Neuroscience, Erasmus MC, P.O. Box 2040, 3000 CA Rotterdam, The Netherlands; ^2^Erasmus University College, Rotterdam, The Netherlands

## Abstract

The present study investigated how gaze following eye movements are affected by stimulus contrast and spatial frequency and by aberrations in central visual acuity due to refractive errors. We measured 30 healthy subjects with a range of visual acuities but without any refractive correction. Visual acuity was tested using a Landolt-C chart. Subjects were divided into three groups with low, intermediate, or good visual acuity. Gaze following responses (GFR) to moving Gabor patches were recorded by video-oculography. In each trial, the subjects were presented with a single Gabor patch with a specific spatial frequency and luminance contrast that moved sinusoidally in the horizontal plane. We observed that GFR gain decreased with increasing spatial frequency and decreasing contrast and was correlated with visual acuity. GFR gain was lower and decreased more for subjects with lower visual acuity; this was especially so for lower stimulus contrasts that are not tested in standard acuity tests. The largest differences between the groups were observed at spatial frequencies around 4 cpd and at contrasts up to 10%. Aberrations in central visual acuity due to refractive errors affect the GFR response depending on the contrast and spatial frequency of the moving stimulus. Measuring this effect may contribute to a better estimate of changes in visual function as a result of aging, disease, or treatments meant to improve vision.

## 1. Introduction

Vision is the ability to observe the world by interpreting light that is reflected from the surroundings and reaches the retina. Loss of visual function can severely affect daily human activities and may effectively decrease the quality of life [1, 2]. Loss of vision can be caused by various ocular diseases, such as retinitis pigmentosa, macular degeneration [3], or glaucoma. Loss of vision can also occur as a symptom of other disorders like multiple sclerosis [4] or diabetic retinopathy.

Visual acuity is one of the important aspects of visual function. Currently, visual acuity is mainly assessed by measuring central visual acuity using, for instance, a letter chart on which a subject has to read increasingly smaller letters while standing at a particular distance (Snellen acuity). Other acuity measures use the E-chart or the Landolt-C chart, in which subjects are asked to report the orientation of a capital E or C, respectively. A person with normal acuity (20/20 vision) can identify the standardized symbols on the chart at a distance of about 6 meters (20 feet). A person with 20/30 vision can identify symbols on the chart from 20 feet that a person with normal acuity could see from 30 feet. This method to assess acuity measures the highest resolution that the visual system can perceive and is useful to determine refractive errors of the eye.

However, several issues arise using such a measure. For instance, these acuity tests often yield highly variable results between examinations due to, for instance, observer-based (i.e., by clinicians or experimenters) variability [5] and differences in recording settings, such as distances from chart and light conditions [6]. In addition, active cooperation of the observer is required: the observer has to actively report, for instance, the orientation of a C. This not only requires compliance of the observer, but also means that it is a subjective measure of visual function, in the sense that no strong conclusions can be drawn from an inability to read out the next line. Finally, with this type of acuity tests only a small subset of the sensitivity of the visual system is probed: objects with high spatial frequencies at very high contrast [7].

Several studies related visual acuity to optokinetic eye movements [8], or smooth pursuit eye movements [9], and did so by varying the spatial frequency of the stimulus. Optokinetic eye movements refer to the following of a moving pattern of dots or stripes in the full visual field. These studies used gaze and ocular following responses as a tool to assess visual function in visually impaired participants and reported that reduced visual acuities decreased the amplitude of the following response. However, these studies did not vary the contrast of the stimulus and, hence, did not determine the interaction between visual acuity and contrast sensitivity in more detail.

Contrast sensitivity is the ability to detect differences in contrast between shades of gray in a visual stimulus. This sensitivity varies with spatial frequency, that is, the level of visual detail of the stimulus. A contrast sensitivity test measures the smallest amount of contrast needed to detect a visual stimulus and provides a more complete quantification of a person's visual capabilities by taking two variables into account, spatial detail and contrast. Contrast sensitivity is measured by asking an observer to detect or discriminate gratings as targets instead of symbols [10, 11]. Sine-wave gratings possess useful mathematical properties and early stages of visual processing are optimally “tuned” to such targets [12, 13]. Each sine-wave grating consists of a given spatial frequency which is specified in terms of the size of the grating at the back of the eye considering the number of sinusoidal luminance cycles per degree (cpd) of visual angle. The contrast of the target grating at a specific spatial frequency is then varied while the observer's contrast detection threshold is determined.

The contrast sensitivity function has become a well-established tool to probe the functional integrity of the visual system [14]. Over the last decades many techniques, including psychophysics [15] and the recording of optokinetic nystagmus [8, 9], have been described to measure the contrast sensitivity function. Leguire et al. [14] compared the contrast sensitivity function based on psychophysics with optokinetic measurements in a small group of healthy subjects and found a good association between the two measures. Here we aim to determine the contrast sensitivity function in humans by measuring the gaze following response (GFR) in reaction to moving stimuli of varying contrasts and spatial frequencies. The GFR is an eye movement that drives the eye to follow stimuli in the visual field and may contain both voluntary and involuntary components. This technique was originally developed to determine contrast sensitivity in mice [16]. The present study is for proof of principle only and shows how foveally induced gaze following responses to moving Gabor patches are affected by contrast and spatial frequency of the stimulus. In addition, we studied how these eye movement responses are affected by aberrations in central visual acuity due to uncorrected refractive errors.

## 2. Materials and Methods

### 2.1. Subjects

We measured 30 healthy subjects (13 females) between 22 and 57 years of age (median of 27 years). One eye was measured per subject without any refractive correction, while the other eye was patched. The study followed the tenets of the Declaration of Helsinki and informed consent was obtained from all subjects before the experiment.

### 2.2. Visual Acuity

Central visual acuity was assessed using a Landolt-C chart, which requires an observer to report the orientation of a gap in a ring. The size of the ring and gap is reduced every three rings. The visual acuity score of the observer was defined by the smallest visual angle of the ring for which the observer reported the orientation correctly at least two times. Variability was limited by using a fixed viewing distance of 6 meters (~20 feet) and by having one experimenter testing all subjects under identical light conditions. Subjects were divided into three groups based on these visual acuity scores: low (score of 20/200 and below), intermediate (scores between 20/200 and 20/20), and high (score of 20/20 and above) visual acuity.

### 2.3. Contrast Sensitivity Measurements

**Figure 1:**
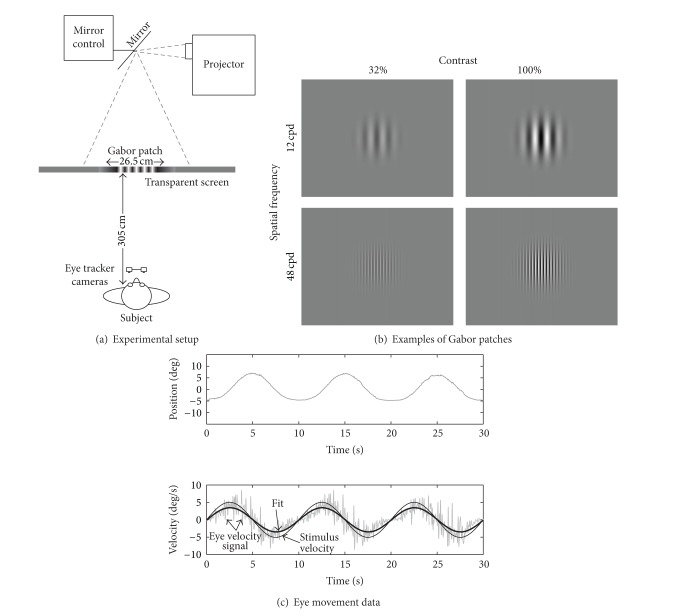
(a) shows a schematic drawing of the experimental setup. The stimulus, a Gabor patch, was back-projected via mirrors on a transparent screen. Movement of the mirrors induced a horizontal movement on the screen. (b) shows four examples of Gabor patches. Each stimulus contained a Gabor patch with a unique combination of spatial frequency and black-white contrast. (c) shows an example of the eye movements of an observer who explicitly followed the movement of the Gabor patch. This observer was not included in this experiment. The Gabor patch moved sinusoidally from left to right in the horizontal plane. The top plot of (c) shows the raw eye position trace. The bottom plot of (c) shows the corresponding eye velocity trace (gray line), the stimulus velocity trace (thin black line), and the fitted sinusoid through the eye velocity signal (thick black line).

All experiments were performed in a darkened room. The gaze following response (GFR) was evoked by moving a visual stimulus horizontally. The stimulus was back-projected via computer-controlled movable mirrors (Laser2000, The Netherlands) on a transparent screen (135 by 99 cm) using a digital projector (Sanyo PLV-Z2) with a resolution of 1024 by 768 pixels (see Figure 1(a)). Viewing distance was 305 cm.

Each stimulus showed a circular Gabor patch with a standard deviation of 5 degrees of visual angle in diameter, thus covering the fovea and a substantial part of the parafoveal area when looked at. The Gabor patches were generated in Matlab (The Mathworks, Natick, MA, USA). A Gabor patch consisted of vertically oriented lines with a particular luminance that was determined by a sinusoid having a specific frequency (0.48, 0.96, 1.93, 3.87, or 7.74 cycles per degree) and a specific black and white contrast (1, 2, 4, 8, 16, 32, 48, 64, or 100%). Luminance of the bright (*L*_max⁡_) and dark stripes (*L*_min⁡_) in the center of the patch was measured with a luminance meter (LS-100; Minolta Camera, Osaka, Japan), after which contrasts were calculated according to the Michelson formula: Contrast = (*L*_max⁡_ − *L*_min⁡_)/(*L*_max⁡_ + *L*_min⁡_). Average luminance was 16 cd/m^2^ in all stimulus conditions. For the stimuli with 100% contrast, the luminance of the dark stripes was 0.15 cd/m^2^ and the luminance of the white stripes was 32 cd/m^2^.

Combining these two variables yielded 45 unique Gabor patches and each was presented once in a random order (see Figure 1(b) for stimulus examples).

The movements of the mirrors were controlled by a computer running Spike-2 (version 4.20, Cambridge Electronic Design) in such a way that the stimulus moved sinusoidally in the horizontal plane (frequency of 0.1 Hz, peak velocity of 5 degrees per second, and peak to peak amplitude of 15.9 degrees) for three full cycles. Hence, each stimulus presentation lasted for 30 seconds. Between stimulus presentations there was a random blank interval of 5–15 seconds. The experimenter told the subject when the next stimulus was about to be presented. Subjects were instructed to keep their eyes open and look at the stimulus, without specific instructions. The whole experiment lasted for about 40 minutes.

Head movements were restrained using a bite board. Monocular eye movements were recorded using an EyeLink infrared camera system (EyeLink 1 Desktop, SensoMotoric Instruments GmbH, Teltow, Germany) at a 250 Hz sampling rate [17]. Calibration and calibration-accuracy validation were performed prior to each experiment using the standard EyeLink routine.

### 2.4. Analysis

Eye movement recordings were analyzed offline using custom-written software in Matlab. The first 3 seconds of data was discarded. Instantaneous eye velocity was calculated by taking the first derivative of horizontal eye position over time. Fast phases (saccades) were removed using a velocity threshold of 10 degrees per second (i.e., twice the stimulus velocity). A sine-wave was fitted to the remaining velocity signal, yielding a fitted peak eye velocity (see Figure 1(c) for an example).

The eye velocity gain was calculated for each subject, contrast, and spatial frequency by dividing the fitted peak eye velocity by the peak stimulus velocity (fixed at 5 deg/s). Per subject, all gains were then normalized by setting the gain obtained in the highest contrast (100%) and lowest spatial frequency (0.48 cpd) to 1.

A multivariate repeated measures analysis (ANOVA) was carried out for the eye movement gains with three factors: “Group” (3 levels: low, intermediate, and high visual acuity), “Contrast” (9 levels), and “Spatial Frequency” (5 levels). Differences between groups were evaluated by subsequent planned comparisons (uncorrected). All statistical analyses were done in SPSS 20.

## 3. Results

In two subjects, not all responses could be reliably measured due to eye tracking failure; their data was discarded. Based on their visual score in the visual acuity test, the remaining 28 subjects were classified as having low (score of 20/200 and below, *N* = 11), intermediate (scores between 20/200 and 20/20, *N* = 10), or high (score of 20/20 and above, *N* = 7) visual acuity. Ages did not differ between the three groups (median test, *P* = 0.75).

Before normalization (see Methods), the gains across all 45 Gabor presentations and all 28 subjects ranged from 0.0 to 0.53 with a grand overall median of 0.25. After normalization, the GFR gains (averaged over the 45 Gabor presentations) ranged from 0.40 to 0.95 across subjects (median of 0.65). There was a positive correlation between acuity scores and the average normalized GFR gains (Spearman correlation of 0.78, *P* < 0.001).

**Figure 2:**
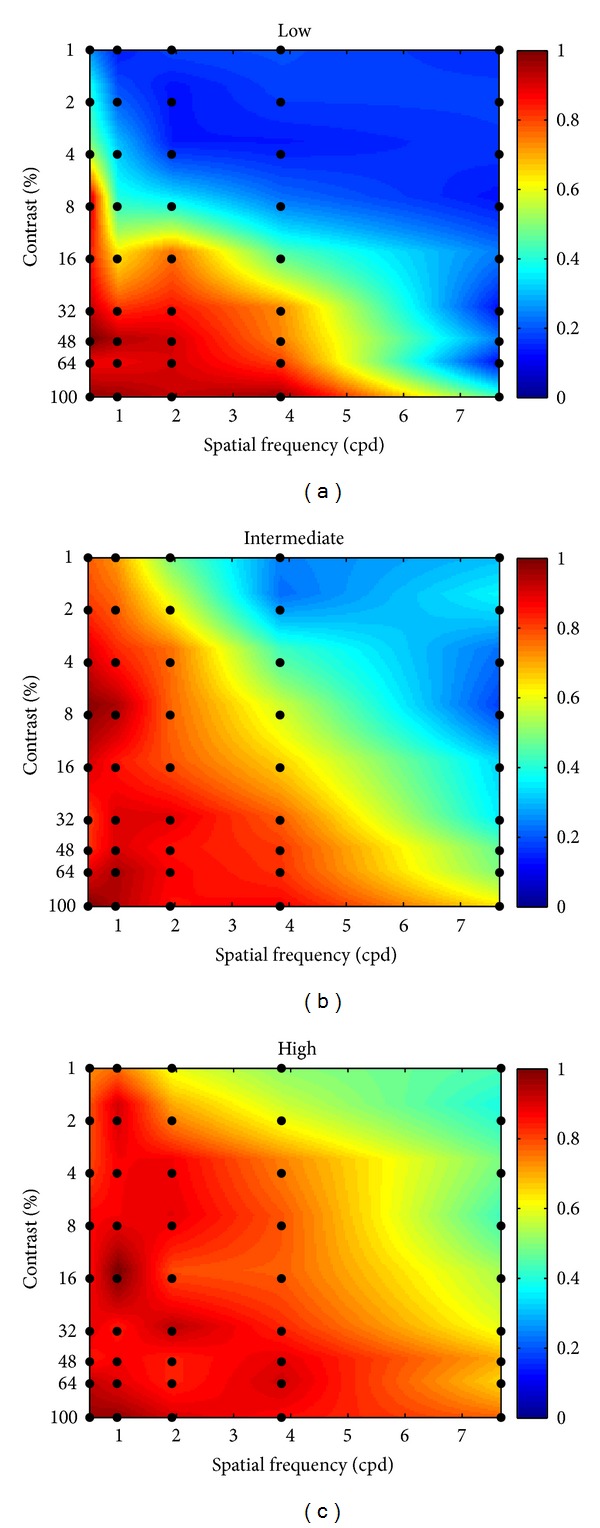
For each of the three groups (low, intermediate, or high visual acuity), the color reflects the averaged normalized GFR gains at 45 combinations of contrast and spatial frequency (indicated by black dots). The space between the measured points is linearly interpolated.

Figure 2 shows the normalized GFR gain as a function of spatial frequency and contrast of the stimulus for the 3 groups separately. Differences can clearly be observed, most prominently at high spatial frequencies and low contrast.

**Figure 3:**
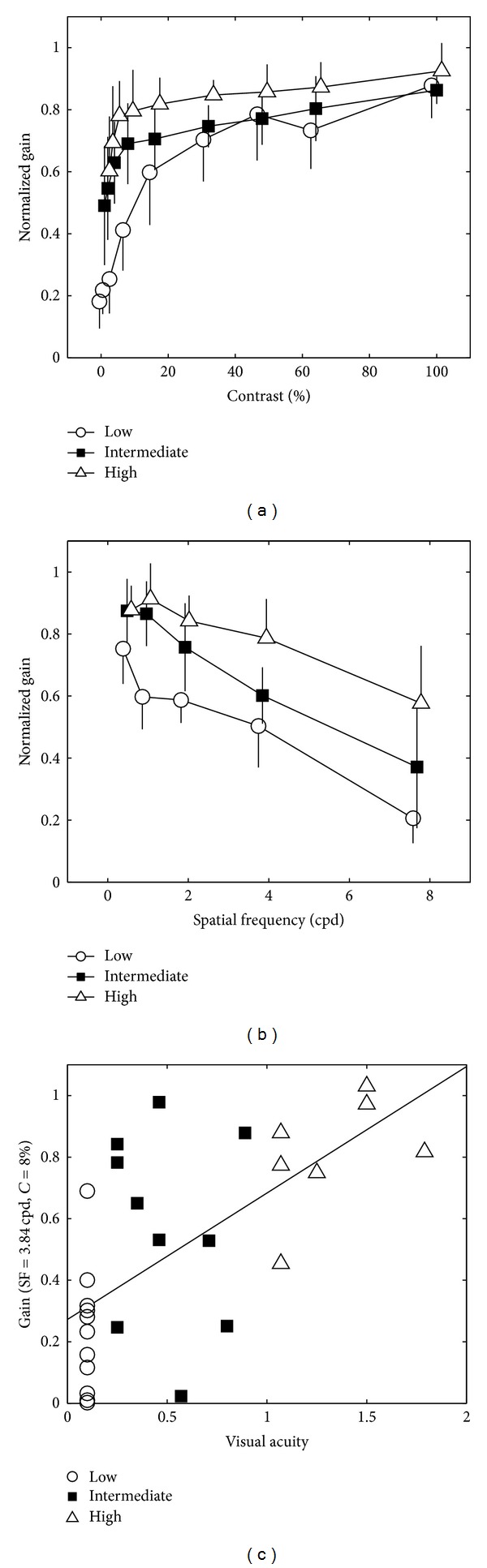
For each of the three groups (low, intermediate, or high visual acuity), the averaged normalized GFR gains are pooled over the 5 levels of spatial frequency and plotted against contrast in (a) and pooled over the 9 levels of contrast and plotted against spatial frequency in (b). The error bars represent standard error of the mean. The normalized GFR gain in response to a moving Gabor patch with a spatial frequency of 3.84 cpd and a contrast of 8% is plotted against the visual acuity score in (c); each symbol represents a single subject.

In order to quantify these differences, the average normalized gains obtained for each contrast and each spatial frequency from each group are plotted in Figures 3(a) and 3(b), respectively.

Statistical analysis showed a significant main effect of “Group” (*F*(2) = 21.4, *P* < 0.001): high acuity subjects had on average higher GFR gains (0.80 ± 0.03 [Standard Error]) than the intermediate (0.69 ± 0.03, *P* < 0.001) and low acuity subjects (0.53 ± 0.03*P* < 0.001). The difference in average gain between the low and intermediate acuity groups was also significant (*P* = 0.024).

The significant main effects of “Contrast” (*F*(8) = 29.5, *P* < 0.001) and “Spatial Frequency” (*F*(4) = 48.4, *P* < 0.001) showed that decreasing the contrast or increasing the spatial frequency of the visual stimulus reduced the GFR gain.

The interactions between “Group” and “Contrast” (*F*(2,8) = 3.62, *P* < 0.001) and between “Group” and “Spatial Frequency” (*F*(2,4) = 3.79, *P* = 0.013) were significant. Post hoc analyses showed that especially for lower contrasts (1%–8%) the low acuity group had significantly lower GFR gains than both the intermediate and high acuity groups (all *P* < 0.01; see Figure 3(a)). The average GFR gain was lowest in the low acuity group for all spatial frequencies. Furthermore, for low spatial frequencies (0.48 to 1.92 cpd) the intermediate acuity group differed from the low acuity group, whereas for higher frequencies (3.84 and 7.68 cpd) the intermediate group differed from the high acuity group (see Figure 3(b)).

From these analyses, we observed that our groups were best separated using a moving Gabor patch with a spatial frequency of about 4 cpd and a contrast of about 8%. We found a positive correlation between visual acuity scores and the normalized GFR gains for this particular Gabor patch (Spearman correlation of 0.64, *P* = 0.002, Figure 3(c)). We did not compute the correlations for the other 44 stimuli.

## 4. Discussion

We observed that the gaze following response (GFR) is influenced by both the spatial frequency and the contrast of a sinusoidally moving sine grating. In addition, this eye movement response was affected by central visual acuity, as it varied between three groups of subjects with varying visual acuity scores. Reduced central visual acuity related to uncorrected refractive errors induced lower GFR gains. This suggests that the GFR response can be used to estimate human contrast-sensitivity function and to determine how well a stimulus is seen.

The method of measuring the GFR has several advantages over a central visual acuity test using, for instance, a letter chart or a Landolt-C chart. For instance, the latter does not cover the whole visual spectrum but only the high spatial frequency and very high contrast ranges [7], and, therefore, a 20/20 result in visual acuity will not always uncover loss of vision for other contrasts and spatial frequencies. Hence, tests in low vision patients (patients with impaired eye sight that cannot be corrected by conventional means) often depend on self-reported changes in visual function and acuity, which can be highly inaccurate [2, 18]. The eye movement response can provide useful additional information, for instance, an extra dimension of data: that of response magnitude. The gaze following responses measured with the contrast sensitivity test are not bimodal (“I can see it” or “I cannot see it,” as in the common acuity test) but graded; the gain of the GFR decreases as stimuli become harder to see [16, 19]. This response sensitivity makes the GFR method suitable to use when screening for small changes in contrast sensitivity, which can be caused by degenerative diseases or by treatment methods that aim to improve visual function in low vision patients. With stimuli covering the whole range of human vision, small changes in contrast sensitivity could be detected using this approach. In addition, using the GFR reflex to test contrast sensitivity provides us with an objective test and without user bias, which often occurs in perception tasks when the stimulus is close to the perceptual threshold.

We tested a small range of spatial frequencies, because there are some technical challenges to measure all the way up to the maximum visible spatial frequency (about 60 cpd [20]) using a standard computer display. For instance, a 24-inch widescreen monitor is approximately 48 cm wide and has a horizontal resolution of 1920 pixels. This means that at most 20 black lines and 20 white lines, each being only 1 pixel wide, can be drawn per cm, resulting in a square wave grating of 20 cycles per cm. At a viewing distance of about 170 cm, this would yield a spatial frequency of 60 cpd. For sine gratings, requiring 4 (or even more) visible lines with different contrasts, the viewing distance has to be doubled (at least) to accommodate the entire stimulus. In our setup, the maximum spatial frequency of projected sine-wave grating was limited by viewing distance (305 cm), screen width (135 cm), and projector resolution (1204 pixels), resulting in a projection of about 41 pixels per degree, that is, a maximum spatial frequency of 10 degrees. However, we already observed a decrease in GFR gain for moderate spatial frequencies up to 10 degrees; higher frequencies would have most likely led to negligible eye movement responses. This observation can be exploited in future studies by modifying the experiment into an adaptive procedure which allegedly restricts the number of required trials, thereby reducing the total time of the test substantially. At present, measuring the GFR at all possible combinations of spatial frequencies and contrasts took about 40 minutes, whereas a regular visual acuity test takes only a few minutes to complete.

In the present study, the nonnormalized GFR gains were rather low. This was probably related to the instruction given to the subject. We told them to look at the stimulus, without explicitly instructing them to fixate at the center of the moving Gabor patch.

Our study suggests that measuring the GFR provides insight into the sensitivity of spatial frequency and contrast in healthy adult subjects. In addition to measuring central visual acuity (preferably by means of a logMAR chart) it may have several clinical applications. For instance, it is known that contrast sensitivity decreases with age [21, 22], which is, however, generally not picked up with an acuity test. In elderly persons, decreased contrast sensitivity contributes to a poor postural stability which leads to a doubling of the already increased likelihood of falling [23]. As a note, the Landolt-C chart measures central visual acuity and we did not assess peripheral visual function. There are many complex ocular conditions; some conditions affect central visual function, like macular degeneration, whereas others result in macular sparing and induce tunnel vision. Moreover, there is a difference between peripheral and central contrast sensitivity. Future studies using the GFR as a measure for contrast sensitivity might use stimuli that target the peripheral retina in particular.

## 5. Conclusion

We conclude that measuring the gaze following response (GFR) for a wide range of stimulus contrasts and spatial frequencies is useful for observing changes in contrast sensitivity. We observed that worse central visual acuity affects this eye movement response. Measuring the GFR may contribute to a better estimate of changes in visual function as a result of ageing, disease, or treatments meant to improve visual function.
